# Erythrocyte Binding Activity Displayed by a Selective Group of *Plasmodium vivax* Tryptophan Rich Antigens Is Inhibited by Patients’ Antibodies

**DOI:** 10.1371/journal.pone.0050754

**Published:** 2012-12-06

**Authors:** Rupesh Kumar Tyagi, Yagya Dutta Sharma

**Affiliations:** Department of Biotechnology, All India Institute of Medical Sciences, Ansari Nagar, New Delhi, India; State University of Campinas, Brazil

## Abstract

*Plasmodium vivax* is a very common but non-cultivable malaria parasite affecting large human population in tropical world. To develop therapeutic reagents for this malaria, the parasite molecules involved in host-parasite interaction need to be investigated as they form effective vaccine or drug targets. We have investigated here the erythrocyte binding activity of a group of 15 different *Plasmodium vivax* tryptophan rich antigens (PvTRAgs). Only six of them, named PvTRAg, PvTRAg38, PvTRAg33.5, PvTRAg35.2 PvTRAg69.4 and PvATRAg74, showed binding to host erythrocytes. That the PvTRAgs binding to host erythrocytes was specific was evident from the competitive inhibition and saturation kinetics results. The erythrocyte receptors for these six PvTRAgs were resistant to trypsin and neuraminidase. These receptors were also chymotrypsin resistant except the receptors for PvTRAg38 and PvATRAg74 which were partially sensitive to this enzyme. The cross-competition studies showed that the chymotrypsin resistant RBC receptor for each of these two proteins was different. Altogether, there seems to be three RBC receptors for these six PvTRAgs and each PvTRAg has two RBC receptors. Both RBC receptors for PvTRAg, PvTRAg69.4, PvTRAg33.5, and PvTRAg35.2 were common to all these four proteins. These four PvTRAgs also shared one of their RBC receptors with PvTRAg38 as well as with PvATRAg74. The erythrocyte binding activity of these six PvTRAgs was inhibited by the respective rabbit polyclonal antibodies as well as by the natural antibodies produced by the *P. vivax* exposed individuals. It is concluded that only selective few PvTRAgs show erythrocyte binding activity involving different receptor molecules which can be blocked by the natural antibodies. Further studies on these receptor and ligands may lead to the development of therapeutic reagents for *P. vivax* malaria.

## Introduction

*Plasmodium vivax* affects millions of people every year worldwide. This parasite remains non-cultivable in the laboratory. In the past, the disease caused by *P. vivax* was considered benign as compared to *P. falciparum*. However, recent reports indicate that, like *P. falciparum,* this parasite can also cause complications and thus increases severity of the disease [1]–[4]. There is also emergence of drug resistance in *P. vivax* [5]–[9]. Therefore, efforts should be made for the development of new therapeutic approaches in order to control this parasitic disease.

Molecules involved in host-parasite interaction are essential for parasite development, so these molecules comprise excellent targets for the development of vaccines and drugs. Several parasite molecules are known to interact with its host cell for various functions such as invasion, pathophysiology or signaling pathways [10]–[12]. *Plasmodium vivax* uses Duffy antigen to invade the human erythrocytes but there are reports which indicate that this parasite may also be using other receptors for this purpose [13]–[15]. Furthermore, a simian malaria parasite *P. knowlesi* which is very close to *P. vivax* also uses Duffy antigen for invasion but can also use other pathways for this purpose [16]. Studies are, therefore, required to identify these additional parasite molecules which are involved in host-parasite interaction.

The tryptophan rich proteins (pypAg-1 and pypAg-3) were first characterized from *P. yoelii* which showed binding to mouse erythrocytes for invasion process [17], [18]. They were found to be immunogenic and mice immunized with these recombinant proteins were protected against *P. yoelii* infection [17]. Homologues of pypAg-1 and pypAg-3 in *P. falciparum* have been identified and named as PfTryThrA (tryptophan-threonine rich antigen) and PfMaTrA (merozoite associated tryptophan rich antigen), respectively [19], [20]. These two *P. falciparum* homologs also show binding to human erythrocytes and peptides derived from PfTryThrA inhibited the merozoite invasion [21]. *Plasmodium vivax,* however, contains a family of tryptophan rich antigens comprising thirty six of these molecules (www.plasmodb.org) [22]. These proteins have high percentage of tryptophan residues which are positionally conserved [23]. Many of them have been immunologically characterized where they induced significant cellular and humoral responses in human individuals, and showed very little genetic polymorphism in parasite population [24]–[30]. One of them has also been shown to bind to human erythrocytes [31]. Therefore, the purpose of this study was to investigate if there are other tryptophan-rich proteins which are also involved in parasite binding to erythrocytes and then be a good target for drugs or vaccines. We selected fifteen of thirty six PvTRAgs for this study as they showed the highest homology with the previously described PvTRAg [27].

**Figure 1:**
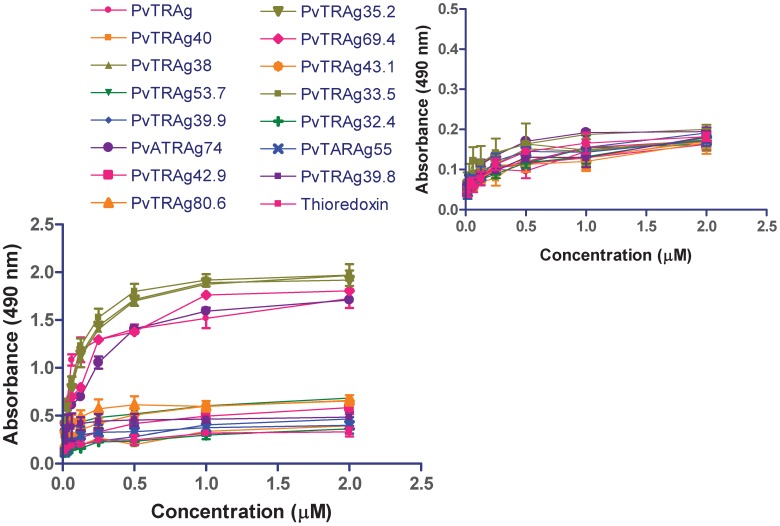
Erythrocyte binding activity of 15 different *P. vivax* tryptophan rich antigens (PvTRAgs) by Cell-ELISA. ELISA plate was coated with erythrocytes or lymphocytes (Fig.1 inset) and reacted with different concentration of Histidine-tagged recombinant proteins, followed by primary anti-his antibody and HRP conjugated secondary antibody. Color was developed with OPD and O.D. was measured at 490 nm. Data shown are the mean ± S.D. of at least 3 independent experiments. Recombinant bacterial thioredoxin was taken as negative control and PvATRAg74 as positive control.

## Materials and Methods

### Ethics Statement

The study was conducted in accordance with the ethical guidelines of the Indian Council of Medical research for collecting the heparinized blood from healthy lab individuals and from the *P. vivax* infected individuals. The written informed consent was obtained from the individuals prior to their blood collection. Ethics committee of All India Institute of Medical Sciences, New Delhi, had approved the study via approval number IEC/NP-342/2012 & RP-11/2012.

**Figure 2:**
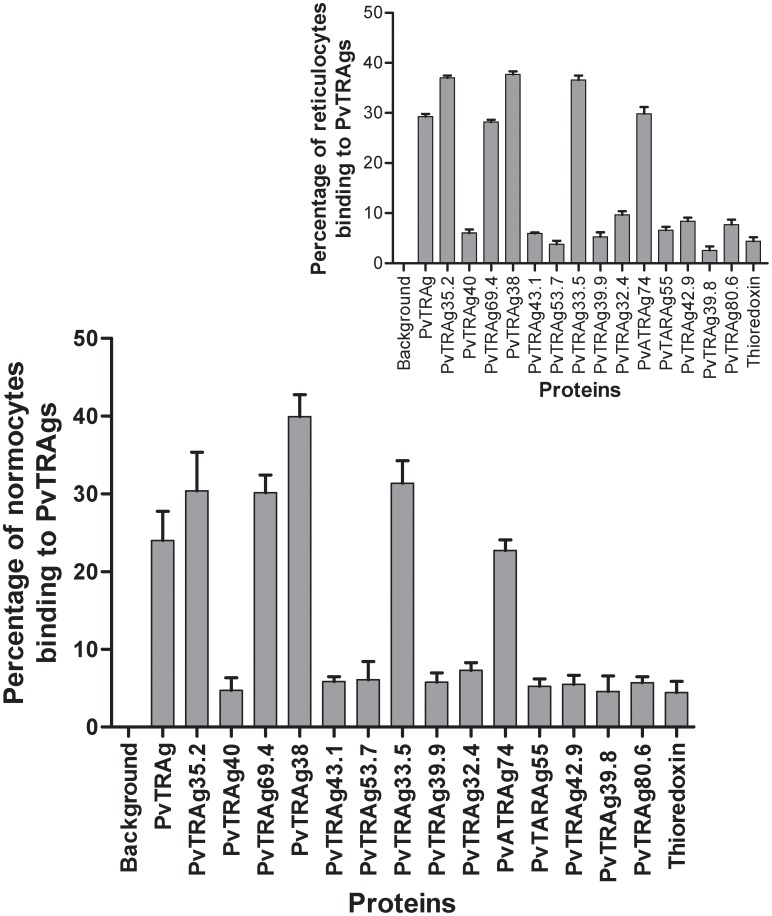
Erythrocyte binding activity of 15 different *P. vivax* tryptophan rich antigens (PvTRAgs) by Flowcytometry. Total erythrocytes were used for the binding assay. Dot plot and Histogram of erythrocytes gates were used for the analysis. Bar diagram shows the binding of all different PvTRAgs with the erythrocytes or reticulocytes (inset). One million RBC were incubated with 1 µM concentration of Histidine-tagged recombinant proteins. Histidine-tagged PvATRAg74 and bacterial recombinant thioredoxin were used as positive and negative controls, respectively. Data shown are the mean ± S.D. of at least 3 independent experiments. The difference between PvTRAgs and thioredoxin binding to erythrocytes was significant (p<0.001). Background, no PvTRAg was added.

### Materials

A total of 15 *P. vivax* tryptophan-rich antigen genes were cloned, expressed in *E. coli* and recombinant proteins were purified. Expression and purification of some of these PvTRAgs have previously been described [24], [26], [28]–[30]. Remaining PvTRAgs were cloned and expressed using the methods described elsewhere [23]. The recombinant proteins expressed in PproExHT vector were purified by Ni-NTA affinity chromatography. Rabbit antibodies against PvTRAgs used here were available in the lab (Antibodies in rabbits were raised by injecting subcutaneously, at multiple sites, with ∼300 µg of each of the purified Histidine-tagged PvTRAg in Complete Freund’s Adjuvant. Pre-immune serum was collected before the first immunization. After 3 weeks of primary immunization, three consecutive boosters were given at 2 week intervals each, with 110 µg of the recombinant protein emulsified in incomplete Freund’s adjuvant. Ten days after the last immunization, immune sera were collected from the blood, aliquoted and stored at −20°C)Previously expressed and purified Histidine-tagged PvATRAg74 [31] and bacterial thioredoxin from *Desulfovibrio desulfuricans* [32] were also available in the lab. Histidine-tagged recombinant *P. vivax* Duffy binding protein region II (PvR II) was a kind gift from Dr Chetan Chitnis [33].

**Figure 3:**
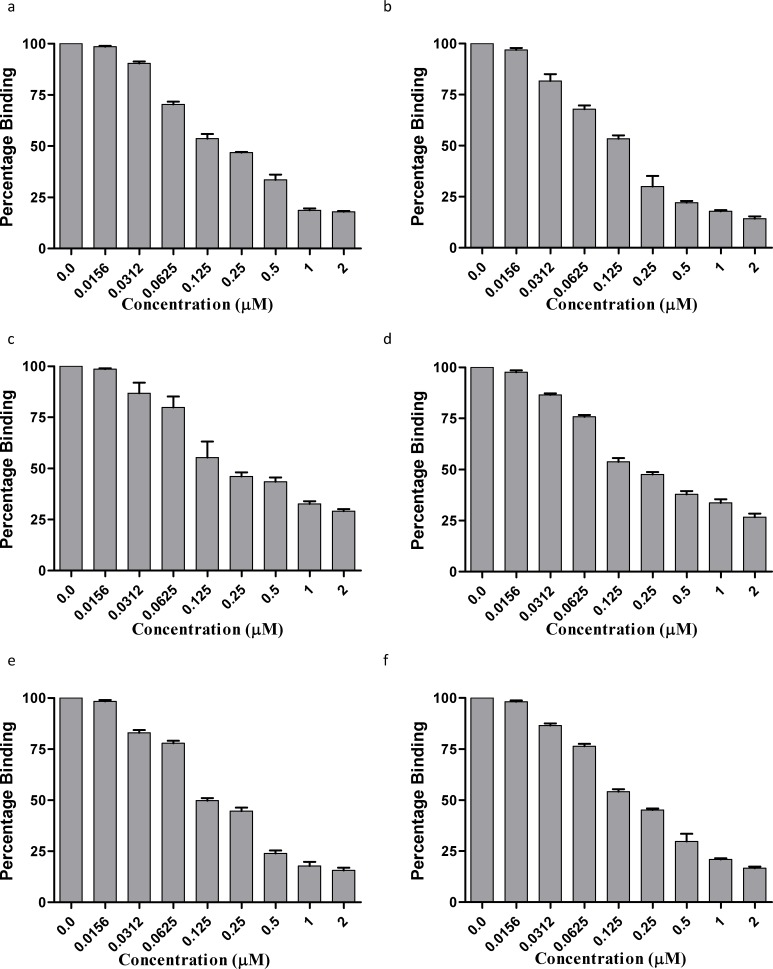
Competitive inhibition of PvTRAgs binding to erythrocytes by **Cell ELISA.** A mixture of Histidine-tagged recombinant PvTRAg (200 nM) and variable amount (0.0 to 2 µM) of respective untagged PvTRAg was added to ∼1 million RBCs. After incubation, the plates were developed by the anti-histidine antibodies as in Fig.1 legend. a, PvTRAg35.2; b, PvTRAg69.4; c, PvTRAg38; d, PvTRAg33.5; e, PvATRAg74; f, PvTRAg. Data shown are the mean ± S.D. of at least 3 independent experiments.

**Figure 4:**
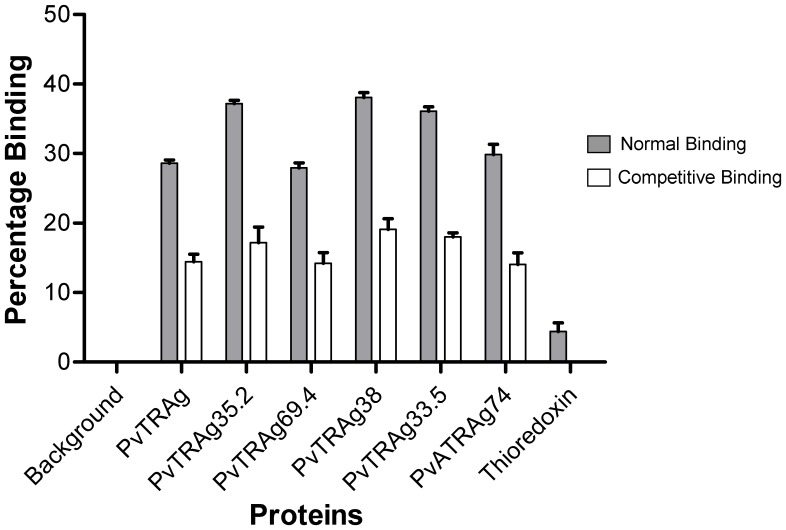
Competitive inhibition of PvTRAgs binding to erythrocytes by Flowcytometry. Equimolar concentration of respective Histidine-tagged and untagged PvTRAg proteins (1 µM each) were added to the (∼1 million) erythrocytes. Competition was not allowed for normal binding, it was used as control. Thioredoxin was used as negative control. Data shown are the mean ± S.D. of at least 3 independent experiments. Background, no PvTRAg was added.

### Erythrocyte Binding Assay

#### Cell – ELISA

ELISA based erythrocyte binding assay was performed according to Alam et al 2008 [31]. Briefly, each well of a 96-well ELISA plate (Corning Incorporation, Corning, NY, USA) was coated with ∼1 million erythrocytes or lymphocytes in duplicate. Next day, plates were blocked with 5% BSA for 2 h at 37°C. After washing, the plates were incubated with different concentrations (15 nM–2 µM) of recombinant proteins for 4 h at room temperature. Plates were washed with PBST (Phosphate Buffer Saline with 0.05% tween 20) and incubated with 1∶2000 dilution of the primary mouse monoclonal anti-His_6_ antibody (Serotech, Raleigh, NC, USA), followed by horseradish peroxidase (HRP) conjugated anti-mouse IgG secondary antibody (Pierce Biotechnology Inc., Rockford, IL, USA). Finally, plates were developed with o-phenyldiamine (OPD) substrate (Sigma-Aldrich, St. Louis, MO, USA) and OD was measured at 490 nm. The recombinant Histidine-tagged PvATRAg74 [31] and bacterial thioredoxin from Desulfovibrio desulfuricans [32] were used as positive and negative controls, respectively.

**Figure 5:**
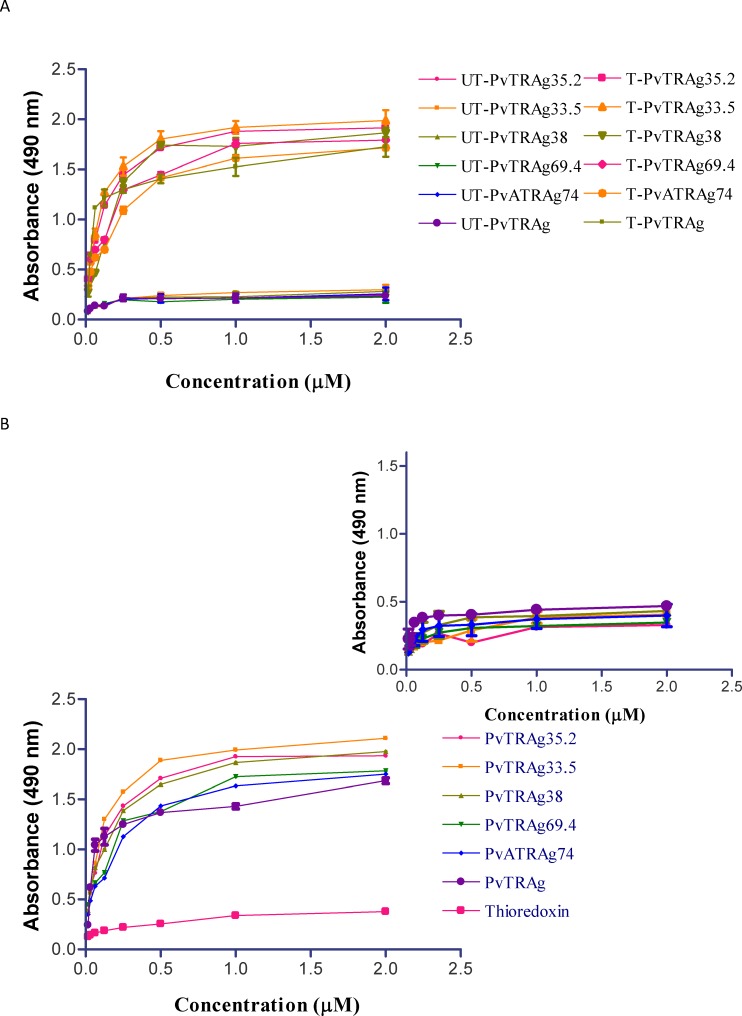
Specificity of PvTRAgs binding to erythrocytes by Cell – ELISA. ELISA plate was coated with one million RBC and incubated with 1 µM of untagged PvTRAgs (A) or untagged thioredoxin (B) followed by incubation with different concentrations of Histidine-tagged recombinant proteins. The plate was developed with anti-histidine antibodies as in Fig.1. In the negative control well, Histidine-tagged thioredoxine was added at different concentrations. For Inset, the ELISA plate coated with one million erythrocytes was incubated with untagged PvTRAgs followed by incubation with Histidine-tagged thioredoxin at different concentrations. Plates were developed as in above. Data shown are the mean ± S.D. of at least 3 independent experiments.

For competitive inhibition studies, the Histidine tag of the recombinant PvTRAgs was first removed by the treatment of AcTEV protease from Invitrogen using the manufacturer’s protocol (Invitrogen Life Sciences, Carlsbad, CA, USA). A fixed amount of Histidine-tagged recombinant PvTRAgs (200 nM) were mixed with increasing concentration (0.0–2 µM) of respective untagged PvTRAg. This mixture was then added to a 96 well ELISA plate already coated with ∼1 million erythrocytes and incubated for 4 h at room temperature. After washing with PBST, plate was processed as above. For positive control, no untagged PvTRAg (only PBS) was pre-incubated with respective Histidine-tagged recombinant PvTRAg.

**Figure 6:**
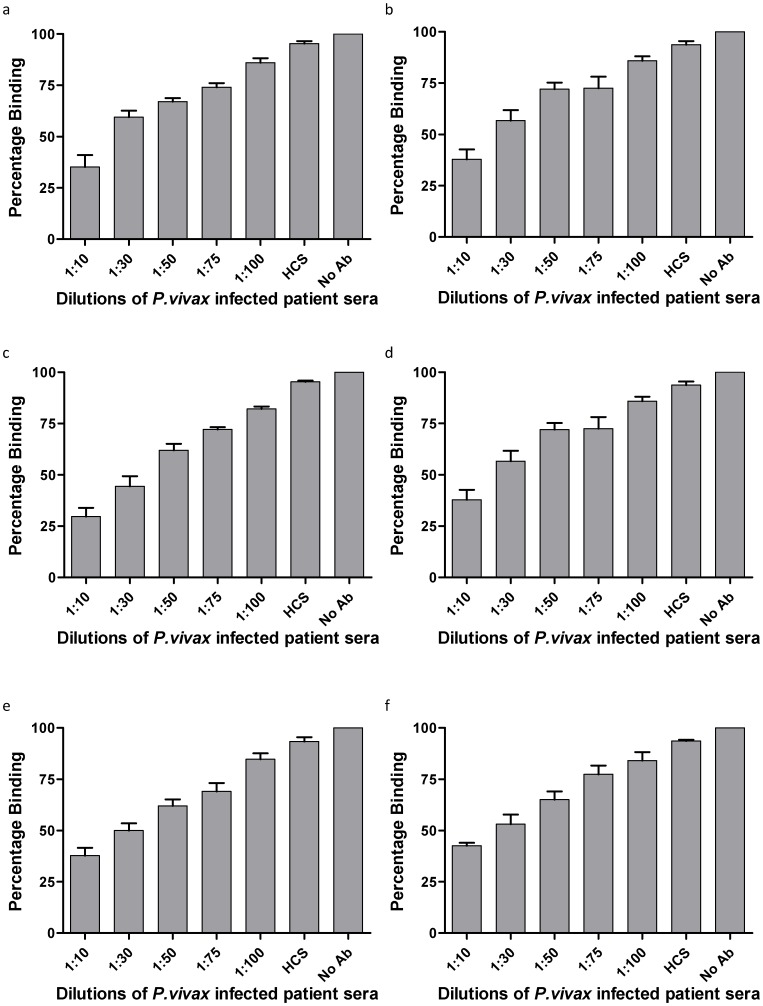
Inhibition of erythrocyte binding activity by patients’ sera. Histidine-tagged PvTRAgs (250 nM) were mixed with different dilutions of *P.vivax* patients’ sera before adding to the microtiter plate coated with one million erythrocytes. Further steps of colour development were same as in Fig.1. a, PvTRAg35.2; b, PvTRAg69.4; c, PvTRAg38; d, PvTRAg33.5; e, PvATRAg74; f, PvTRAg. Data shown are the mean ± S.D. of at least 3 independent experiments. No Ab, no antibody but only 1×PBS; HCS, healthy control sera from humans.

**Figure 7:**
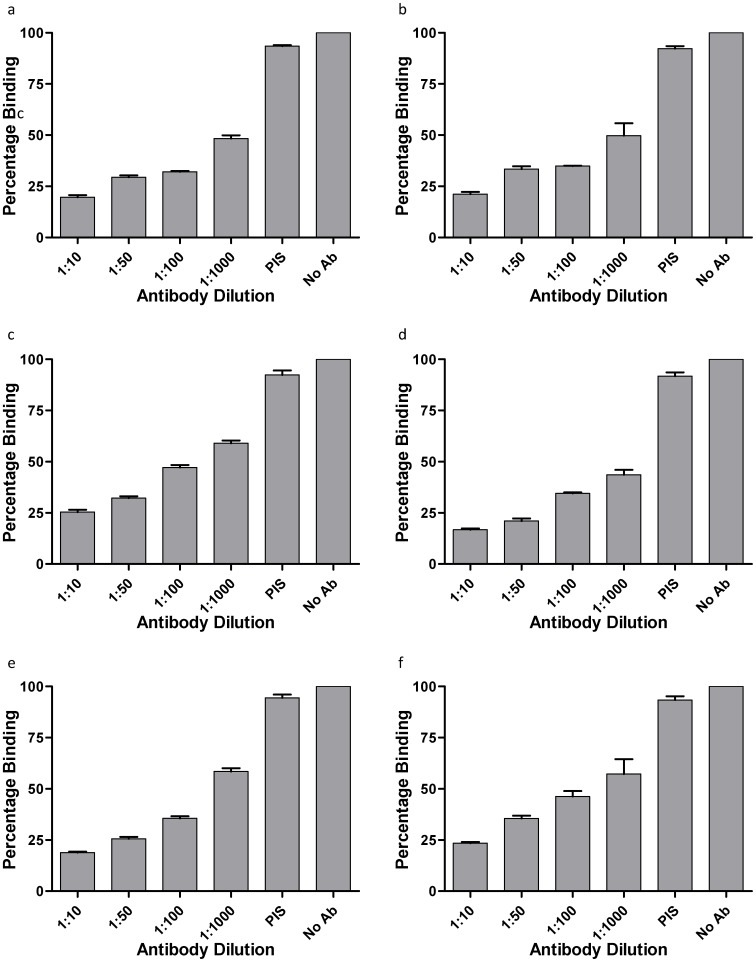
Inhibition of erythrocyte binding activity by rabbit anti-PvTRAgs antibodies. Histidine-tagged PvTRAgs (250 nM) were mixed with different dilutions of polyclonal antisera raised in rabbit against respective PvTRAg before adding to the microtiter plate coated with erythrocytes. Further steps of colour development were same as in Fig.1. a, PvTRAg35.2; b, PvTRAg69.4; c, PvTRAg38; d, PvTRAg33.5; e, PvATRAg74; f, PvTRAg. Data shown are the mean ± S.D. of at least 3 independent experiments. No Ab, no antibody but only 1×PBS; PIS, pre-immune sera.

**Table 1:** Erythrocyte binding inhibition of PvTRAg38 by purified antibodies from the *P. vivax* patients’ sera.

Dilutions of Antibody	Erythrocyte Binding Inhibition	
	Before Antibody Purification (%)	After Antibody Purification* (%)
1∶10	62.5±4.2	84.5±4.3
1∶30	58.7±3.4	79.7±3.9
1∶50	30.6±4.1	73±3.2
1∶75	28.4±5.1	62.2±3.4
1∶100	15.6±3.3	59.3±4.6

Values are mean ± standard deviation of three different experiments. The erythrocyte binding inhibition increased significantly after antibody purification (P<0.05).

Normal Healthy Sera at 1∶10 dilutions showed 4.1±2.3% erythrocyte binding inhibition.

Erythrocyte binding without antibody (only PBS) was considered as 100% binding.

* Antibodies were affinity purified from the pooled *P.vivax* infected patient sera using CarboxyLink™ Immobilization Kit (Thermo scientific, Rockford, USA) according to manufacturer’s protocol. Briefly, 2 mg of PvTRAg38 was coupled to the resin, and after washing with PBS, bound antibody was eluted by 0.2 M glycine-HCl. The purified antibody was dialysed in 50 mM Tris-HCl and used in the erythrocyte binding inhibition assay.

**Table 2:** Cell-ELISA based cross-inhibition of PvTRAgs binding to erythrocytes by rabbit antibodies raised against the same antigen or against its close homolog.

Antigens	Erythrocyte Binding Inhibition*	
	Self Antibody%±S.D.	Homolog Antibody %±S.D
PvTRAg38	52.4±2.3	31±1.6 (PvATRAg74)
PvATRAg74	64.8±0.8	43.6±2.2 (PvTRAg38)
PvTRAg33.5	64.5±0.7	41.8±6.9 (PvTRAg35.2)
PvTRAg35.2	66.8±0.7	42.6±1.0 (PvTRAg33.5)
PvTRAg	52.9±2.6	34.2±0.6 (PvTRAg69.4)
PvTRAg69.4	63.5±0.3	42.9±5.6 (PvTRAg)

* In a cell-ELISA assay, the Histidine tagged PvTRAgs were incubated with polyclonal antisera raised in rabbits at 1∶100 dilutions against the same antigen or its closest homolog (shown in brackets). Pre-incubated mixture was then allowed to react with erythrocytes and binding was detected by monoclonal anti-His_6_ antibody as described in the text. Binding of PvTRAgs in the absence of antibody is taken as100%. Values are mean ± standard deviation of three different experiments. The erythrocyte binding inhibition between self antibody and homologue antibody was significantly different (P<0.05).

For saturation binding study, each of the untagged PvTRAg protein at 1 µM concentration was added to the already coated 96 well ELISA plate with ∼1 million erythrocytes and incubated for 4 h at room temperature. Plate was washed thrice with PBS and incubated for 3 h at room temperature with increasing concentration (15 nM –2 µM) of Histidine-tagged PvTRAg proteins. For positive control, coated erythrocytes were not pre-incubated with untagged PvTRAgs. The plates were incubated with mouse anti-His_6_ monoclonal primary antibody and processed for color development, as described above. In another set of experiment, 1 µM of untagged thioredoxin protein was added to the already coated 96 well ELISA plate with ∼1 million erythrocytes and incubated overnight. Then plate was incubated with increasing concentration (15 nM–2 µM) of Histidine-tagged recombinant PvTRAgs and processed further, as described above, using mouse anti-His_6_ monoclonal antibody. In yet another experiment, 1 µM of untagged PvTRAg proteins were pre-incubated with ∼1 million erythrocytes in ELISA plate. Then Plate was incubated with increasing concentration (15 nM–2 µM) of Histidine-tagged recombinant thioredoxin. The plate was processed as described above.

**Figure 8:**
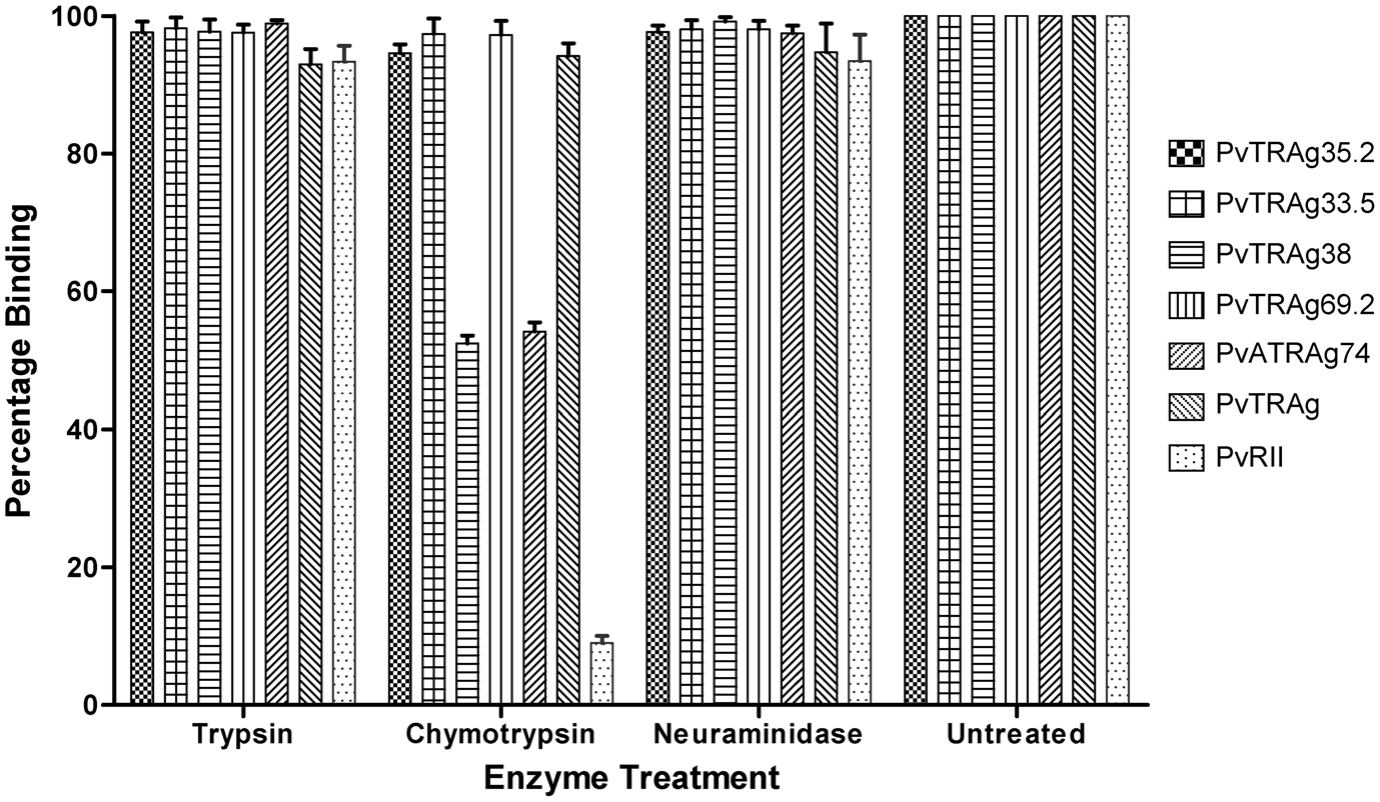
Effect of enzyme treatment of erythrocytes on their binding capacity to PvTRAgs. Human erythrocytes were pretreated with Trypsin, Chymotrypsin or Neuraminidase prior to their coating to the microtiter plate. Then Histidine-tagged recombinant PvTRAgs (1 µM) or *P.vivax* duffy binding protein region II (PvRII) were used for the binding assay with these treated RBCs using the same steps as in Fig1. Untreated RBCs were taken as controls. Data shown are the mean ± S.D. of at least 3 independent experiments.

#### Flow Cytometry

The binding assay was performed according to Tran et al [34]. Briefly, ∼1 million human erythrocytes were incubated for 4 h at room temperature with 1 µM Histidine-tagged recombinant PvTRAg proteins. Erythrocytes were pelleted by centrifugation at 2000×g for 5 minutes. Pellets were washed two times with 1% BSA in PBS and incubated with mouse anti-penta-His Alexa Fluor 647 conjugated mAb (Qiagen, Gmbh, Hilden, Germany) for 1 h at 4°C in the dark. Samples were again pelleted and washed three times with 1% BSA in PBS and incubated with Thiazole Orange (TO) Retic-COUNT reagent (Becton-Dickinson Co. San Jose, CA, USA) for 45 minutes at 25°C. After washing the pellet, it was suspended in 1% BSA (in PBS) for acquiring. Two hundred thousand total events were acquired per sample using A BDLSRII Flow Cytometer (Becton Dickinson Immunocytometry Systems, Palo Alto, CA, USA) by using Facs Diva software (Dot plot showing gating strategy is given in **Figure S1**). The recombinant Histidine-tagged PvATRAg74 [31] and bacterial thioredoxin from *Desulfovibrio desulfuricans* [32] were used as positive and negative controls, respectively.

For competitive inhibition studies, ∼1 million human erythrocytes were incubated with 1 µM of Histidine-tagged PvTRAgs and 1 µM of respective untagged PvTRAgs at room temperature for 4 h. After washing with 1% BSA in PBS, the samples were processed further as described above using mouse anti-penta-His Alexa Fluor 647 conjugated mAb (Qiagen, Gmbh, Hilden, Germany).

### Antibody Inhibition of PvTRAgs Binding to Human Erythrocytes

Each of the Histidine-tagged PvTRAg (250 nM) was pre-incubated with different dilutions (1∶10, 1∶50, 1∶100 and 1∶1000) of polyclonal sera (raised in rabbit against each of the PvTRAg) for overnight at 4°C. This recombinant PvTRAg and anti-PvTRAg antibody reaction mix was then allowed to bind to ∼1 million erythrocytes in a 96 well ELISA plates. Plates were then processed for color development as described above after exposure with mouse anti-His6 monoclonal antibody. Sera from three different *P. vivax* exposed individuals were also used at different dilutions (1∶10, 1∶30, 1∶50, 1∶75 and 1∶100) for the inhibition of PvTRAgs binding to erythrocytes. No antibody (only PBS) and pre-immune rabbit sera or sera from healthy uninfected individuals were taken as positive control.

### Enzyme Treatment of Human Erythrocytes

Five percent haematocrit-adjusted blood samples in the PBS buffer were treated separately with 1 mg/ml final concentration of trypsin or chymotrypsin or with 66 mU/ml of neuraminidase for 1 h at 37°C (Sigma Aldrich, St. Louis, MO, USA). After centrifugation at 2000×g for 5 min, the pellet was washed five times with PBS containing 0.1 mM PMSF. The 96 well ELISA plate was then coated with enzyme treated ∼1 million erythrocytes. For positive control, the ELISA plate was coated with erythrocytes without enzyme treatment. Then the binding assay was performed, as above, with 1 µM concentration of Histidine-tagged recombinant PvTRAgs, *P. vivax* Duffy binding protein region II (PvR II) and bacterial thioredoxin.

### Statistical Analysis

The statistical significance of mean fluorescence intensities (MFIs) were analyzed by unpaired and paired Student’s t test. Calculations were performed using STATA software. Differences were considered significant at P<0.05.

## Results

### PvTRAgs Bind to the Human Erythrocytes

Fifteen different *P. vivax* tryptophan rich antigens (PvTRAgs) were checked for their binding activity to human erythrocytes. Out of these fifteen PvTRAgs, only six of them (PvTRAg, PvATRAg74, PvTRAg33.5, PvTRAg35.2, PvTRAg38 and PvTRAg69.4) showed binding to the human RBCs (**Fig. 1**). PvATRAg74 was used here as a positive control since it had already been shown to contain erythrocyte binding activity [31] and bacterial thioredoxin [32] was used as a negative control which does not show binding to human erythrocytes. None of these PvTRAg proteins showed binding to the lymphocytes (**Fig. 1** **inset**). The PvTRAgs binding to erythrocyte was concentration dependent. This binding increased sharply from 15 nM to 1 µM concentration of PvTRAgs and reaches a plateau thereafter at higher concentrations. Remaining PvTRAgs showed similar pattern as of thioredoxin (**Fig. 1**).

The erythrocyte binding activity of these recombinant PvTRAgs was also confirmed by the flowcytometric analysis. The above mentioned six PvTRAgs which showed erythrocyte binding activity by Cell-ELISA, also showed binding activity by flowcytometry (**Fig. 2**). Their binding activity was significantly higher (p<0.05) than bacterial thioredoxin. These recombinant proteins showed binding to both erythrocytes and reticulocytes. Rest of the nine recombinant PvTRAgs did not show binding to either reticulocytes or erythrocytes, as their binding was similar to that of the negative control thioredoxin. The mean fluorescence intensity (MFI) of reticulocytes and normocytes were also not significantly different for any of the PvTRAgs (**Table S1**). However MFI values for the six of the binder proteins were significantly higher than the remaining nine non binder proteins as well as bacterial thioredoxin.

### PvTRAgs Show Specific Binding to Human Erythrocytes

The six recombinant PvTRAgs, which showed binding to human erythrocytes in the above mentioned experiments, were subjected to competitive inhibition for determining the specificity of binding. For this purpose, the competition was allowed between untagged and Histidine – Tagged PvTRAgs. Results showed that the binding of PvTRAgs decreased with the increased concentration of untagged protein (**Fig. 3**). This is because the respective Histidine-tagged recombinant PvTRAg competes with its untagged protein for erythrocyte binding. The 50% binding was seen at equimolar concentration of tagged and untagged proteins. At higher concentration of untagged proteins, the binding decreased further (**Fig. 3**). The non binder protein PvTRAg40 and bacterial thioredoxin did not compete with the binding of any of the six binder proteins (Data not shown). The binding of recombinant PvTRAgs to total erythrocytes were also found to be decreased to 50% at equimolar concentration of untagged and tagged proteins during flowcytometric analysis (**Fig. 4**). Thus both the experiments confirmed that these PvTRAgs were binding specifically to the erythrocytes.

As mentioned above, the recombinant PvTRAgs were binding to the human erythrocytes in a concentration dependent manner where binding activity increased up to 1 µM concentration of the protein but thereafter it reaches a plateau (**Fig. 1**). This indicated that 1 µM concentration of the protein was saturating all the binding sites of ∼1 million RBCs. Therefore, to check further the specificity of PvTRAgs binding to human RBCs, we saturated all the binding sites on erythrocytes (∼1 million) by respective untagged protein at a 1 µM concentration and then allowed different concentrations of the Histidine-tagged PvTRAgs to bind to them. Results showed that 1 µM of untagged protein indeed was able to saturate all the biding sites in these fixed number of erythrocytes whereas in case of the respective control well, where no untagged PvTRAgs protein was pre-incubated (only PBS was used), the same number of RBCs showed concentration dependent binding to Histidine-tagged PvTRAgs (**Fig. 5**). The specificity of PvTRAgs binding to RBC was proven further by yet another experiment where 1 µM of untagged thioredoxin was pre-incubated with same number of RBCs (∼1 million) prior to incubation with Histidine-tagged PvTRAgs. This bacterial thioredoxin did not bind to RBC in any of these experiments (**Fig. 5**). This shows that the PvTRAgs bind to the human erythrocytes irrespective of the presence of the other non-binding proteins such as bacterial thioredoxin. The specificity was also determined by another assay where untagged PvTRAgs were used to block the coated erythrocytes and then reacted with the Histidine-tagged recombinant thioredoxin (**Fig. 5** **inset**).

### Blocking of PvTRAgs Binding Sites on Host Erythrocytes by Anti - PvTRAgs Antibodies

Different dilutions of *P. vivax* patients’ sera or the rabbit polyclonal sera against each of the PvTRAgs were incubated with individual recombinant antigen at different dilutions before adding to the coated erythrocytes. The individual *P. vivax* exposed patient’s sera inhibited the erythrocyte binding in a dilution dependent manner (**Fig. 6**). This inhibition was found to increase further when we used the purified antibodies from patients’ sera (**Table 1**). These results indicate that antibodies produced against each of the six PvTRAgs during natural course of infection were able to inhibit the binding of these PvTRAgs to the host RBC. Similar results were obtained when rabbit antibodies against each antigen were used as these antibodies also inhibited the binding of its respective antigen to the host erythrocytes (**Fig. 7**). Level of antibody blocking varied from protein to protein. This blocking was also different for rabbit polyclonal antibodies and patients’ sera where latter showed lower level of inhibition **(** **Fig. 6** and **7** **)**. But the purified antibodies from the patients’ sera gave almost similar results as rabbit sera (**Table 1**). The specificity of binding of human antibodies to these PvTRAgs was also confirmed by competing it with rabbit antibodies and vice versa (**Fig. S2**). It was also confirmed by depleting the patients’ sera against the respective antigen. The individual PvTRAg was able to deplete its inhibitory antibodies from the patients’ sera as well as reduced the level of inhibitory antibodies against its close homolog (Data not shown). This indicated the presence of some cross-reacting antibodies in the sera against these PvTRAgs. This was further confirmed by cross-inhibition of erythrocyte binding to PvTRAgs up to certain extent by the rabbit antibodies against its close homolog (**Table 2**).

### Effect of Enzyme Treatment on Host RBC Receptors for PvTRAgs

Parasite molecules are known to bind to different erythrocyte receptors which are known to show different sensitivities towards various enzymes [35]–[38] **.** Thus by abolishing the binding affinity of erythrocytes to PvTRAgs by enzyme treatment, one could tentatively predict the nature of the RBC receptor for the respective protein. We therefore determined the effect of trypsin, chymotrypsin and neuraminidase enzymes on RBCs towards its binding capacity for PvTRAgs. The binding activity of PvTRAg38 and PvATRAg74 were reduced to about half if chymotrypsin treated erythrocytes were used for the assay (**Fig. 8**). But the binding activity of the positive control PvRII [33] was almost completely abolished to these chymotrypsin treated erythrocytes. These values of PvRII were same as the negative control of bacterial thioredoxin. These results indicate that the erythrocyte binding activity of PvTRAg38 and PvATRAg74 is partially affected by chymotrypsin while this enzyme did not have any effect on the RBC receptors for other four PvTRAgs (**Fig. 8**). Even more, the binding activity of none of these six recombinant PvTRAgs was affected if trypsin and neuraminidase treated human erythrocytes were used for binding assay although all the three enzymes were active on their substrates.

### Sharing of RBC Receptors by Different PvTRAgs

**Figure 9:**
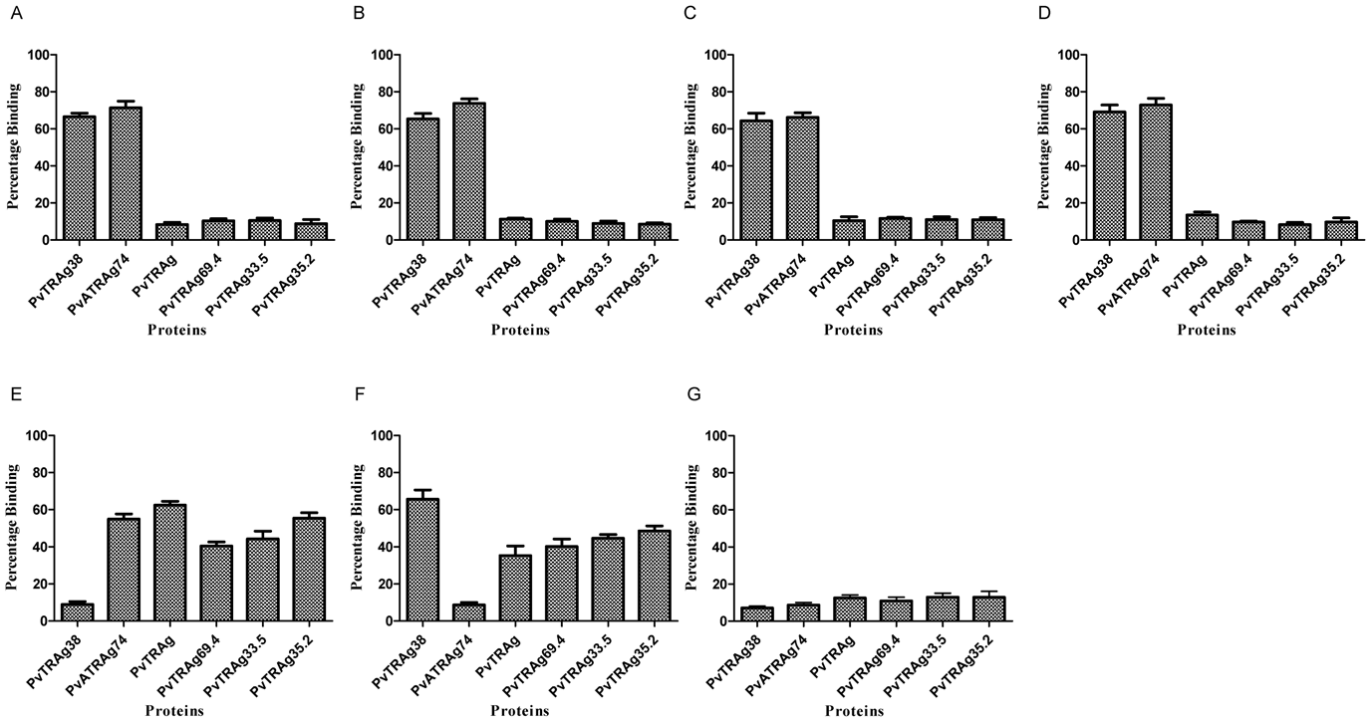
Sharing of RBC receptors by different PvTRAgs. Microtiter plate coated with ∼1 million erythrocytes was incubated with 1 µM of untagged PvTRAg (A), PvTRAg69.4 (B), PvTRAg35.2 (C), PvTRAg33.5 (D), PvTRAg38 (E), PvATRAg74(F), or PvTRAg38 plus PvATRAg74 (G). After washing with PBS, 1 µM of Histidine tagged PvTRAgs were added and binding was detected by monoclonal anti-His_6_ antibody as described in text. Mean ± SD value of percent binding of three different experiments is shown. Binding of PvTRAgs without competitor was considered as 100%.

We performed a cross-competition assay where the erythrocytes were allowed to react with one of the untagged PvTRAg followed by its reaction with Histidine-tagged PvTRAgs. The results are shown in **Figure 9** where four of these proteins (PvTRAg, PvTRAg69.4, PvTRAg33.5, and PvTRAg35.2) completely cross-inhibited erythrocyte binding activity of each other. These four proteins were also cross-inhibiting the erythrocyte binding activity of PvTRAg 38 and PvATRAg74 up to certain extent thus may be competing with one of their RBC receptor. Although PvTRAg 38 and PvATRAg74 share the chymotrypsin sensitive RBC receptor (**Fig. 8**), they did not show complete cross-inhibition of each other indicating that there could be yet another RBC receptor which is different for each of these two proteins. But partial cross-inhibition of this RBC receptor by the other four PvTRAgs indicates that these respective RBC receptors for PvTRAg 38 and PvATRAg74 (chymotrypsin resistant) are shared by these other four PvTRAgs. This was confirmed by the experiment where untagged PvTRAg38 and PvATRAg74 both together blocked binding of these four PvTRAgs with erythrocytes (**Fig. 9G**). Therefore, in total, there could be three different RBC receptors and each PvTRAg will have two of them.

## Discussion

*Plasmodium vivax* contains the largest number of tryptophan-rich antigens, called PvTRAgs, than any other human malaria parasite. In the present study, we have attempted the functional characterization of 15 of these proteins by investigating their capacity to bind to the host erythrocytes. We observed that only six of these 15 PvTRAgs contained the erythrocyte binding activity. That this binding activity was specific was proven by the competition assay as well as by saturation kinetics analysis, both producing the same results. The erythrocyte binding activity of each PvTRAg protein was inhibited by the respective polyclonal sera raised in rabbit or by the *P. vivax* exposed patients’ sera.

These *Plasmodium vivax* tryptophan-rich proteins are expressed during blood stages of the parasite [24]–[27], [29]. Some of them, e.g. PvTRAg, PvTRAg 33.5, and PvTRAg38, are known to be transported to the membrane of the infected erythrocyte or secreted in to the medium outside while status of remaining PvTRAgs remains to be investigated [[27], Unpublished data]. Such proteins will have the possibility of interacting with other host cells such as uninfected RBCs, endothelial cells, and cells in placenta [39], [40]. Our data suggests that these PvTRAgs are interacting with the host erythrocytes and not to the lymphocytes (**Fig. 1** **inset**). Binding of PvTRAgs to the RBCs may suggest that these proteins could be involved in rosette formation where infected RBC can bind to several uninfected erythrocytes. Rosetting may lead to disease pathogenesis [41]. Furthermore, our results have shown that these PvTRAgs were able to bind equally to erythrocytes and reticulocytes (**Fig. 2**). Since, *P.vivax* prefers to invade reticulocytes, it is possible that some of these PvTRAgs are involved in invasion process. It is known that the invasion of human RBC by the parasite is a very complex process [10], [11]. It requires interaction of several parasite molecules to the host erythrocytes for invasion [42]–[45]. Thus the participation of some of the PvTRAgs in this phenomenon can not be ruled out at present. There are evidences that proteins containing tryptophan rich domains are involved in the infectivity of various pathogens like HIV [46], [47] since they provide opportunity for protein-protein interaction [48], [49]. Also the proteins containing tryptophan rich domains are involved in the membrane association [50], signaling pathways [51], and transcriptional activity [52].

Similar to the *P.falciparum* malaria, there are reports showing complications in *P.vivax* patients with organ failure [53]–[57]. This indicates that *P.vivax* should also show cytoadherence to endothelial cells of these internal organs to give rise to such complications. The recent literature indeed reports the binding of *P.vivax* infected erythrocytes to the vascular endothelial cells and to the cells in the placenta [39], [40]. Some of the host and parasite molecules involved in these interactions have been identified such as VIR proteins of the parasite (encoded by *vir* genes) and intracellular adhesion molecule 1 (ICAM-1) and chondroitin sulfate A (CSA) of the host [39], [40]. However, there is an indication that there may be more parasite molecules involved in such endothelial cell type of cytoadherence since VIR only mediated this in part. In this context, the PvTRAgs may also be involved in this type of cytoadherence and thus playing important role in pathophysiology of this parasitic disease. Further studies are required to provide the evidence to this.

The different pattern observed for the different PvTRAgs against their binding activity towards the enzyme treated erythrocytes indicates that different PvTRAgs possibly bind the erythrocyte by more than one RBC receptor (**Fig. 8**). For example, PvTRAg38 and PvATRAg74 may have one common RBC receptor which is different from that of the receptor(s) for the remaining four PvTRAgs. This is because one of the RBC receptor for PvTRAg38 and PvATRAg74 was sensitive to chymotrypsin treatment (**Fig. 8**). On the other hand, the RBC receptor(s) for remaining four PvTRAgs were different as they were resistant to this enzyme. The RBC receptors for all the six PvTRAgs were sialic acid independent and other than glycophorins as they were resistant to neuraminidase and trypsin, respectively.

Chymotrypsin sensitive RBC receptor for PvTRAg38 and PvATRAg74 could be similar to that of the receptor for *P. falciparum* merozoite surface protein 1 (PfMSP1). This is because Band-3 on the erythrocyte surface binds to the PfMSP1 and it is trypsin and neuraminidase resistant but chymotrypsin sensitive [36]. Indeed our unpublished data had confirmed it where we have seen binding of PvTRAg38 and PvATRAg74 to the purified Band-3 protein (Alam and Sharma, unpublished data). It should be noted here that the chymotrypsin treatment of erythrocytes could only abolish ∼50% of its binding activity towards PvTRAg38 and PvATRAg74, unlike the PvRII whose binding activity was almost completely abolished by this enzyme (**Fig. 8**). This also indicates that there may be more than one receptor for each of these two proteins, PvTRAg38 and PvATRAg74, on the host erythrocyte. One of the host receptor may be Band-3, but not Duffy antigen.

Our cross-competition results (**Fig. 9**) revealed the presence of at least three different erythrocyte receptors for these six PvTRAgs and each PvTRAg having two RBC receptors. As mentioned above, one RBC receptor (chymotrypsin sensitive) for PvTRAg38 and PvATRAg74 may be same, the other receptor for each of these two proteins is different. However, each of the second RBC receptor for these two proteins is shared by the receptors for the remaining four PvTRAgs (PvTRAg, PvTRAg69.4, PvTRAg33.5, and PvTRAg35.2). The data also indicates that these four PvTRAgs have two RBC receptors which are common to all four of them. These PvTRAgs thus share one or both of their RBC receptors with each other. Presence of different receptors for different PvTRAgs could also be an indication that these parasite proteins are probably playing different roles during *P. vivax* infection.

We have shown here that binding of PvTRAgs to the human erythrocytes is inhibited by the natural antibodies produced by the infected host during *P. vivax* malaria as well as by the rabbit antibodies specifically raised against these antigens (**Fig. 6** and **7**). These results are important from the view point of developing therapeutic agents to control the disease. The inhibition of erythrocyte binding by the antibodies also indicates that each protein may contain overlapping regions of the antigenic epitopes and erythrocyte binding domains.

We conclude here that not all but only a selective few PvTRAgs bind to the human erythrocytes. The binding is very specific and it is inhibited by the antibodies produced against these proteins during natural course of *P. vivax* infection. Furthermore, there seems to be more than one RBC receptor for these six PvTRAgs. PvTRAg38 and PvATRAg74 proteins seem to have two erythrocyte receptors, and Band-3 may be one of them. Other four PvTRAgs also seems to have two common RBC receptors where one is shared by PvTRAg38 and other by PvATRAg74. Further studies are in progress to investigate the respective RBC receptors for these PvTRAgs and their role in erythrocyte invasion process.

## Supporting Information

Figure S1 **Dot plots for binding of PvTRAgs to erythrocytes.** Erythrocytes were incubated with 1 µM of recombinant PvTRAgs and then labeled with an anti-penta-His mAb Alexa Fluor 647 conjugate and Thiazole Orange. A: Unstained, B: Background control without proteins, C: PvTRAg38, D: PvTRAg40 and E: Thioredoxin
(DOCX)

Figure S2 **Cell-ELISA based competition between rabbit antibodies and** ***P.vivax*** **patients’ sera for inhibition of erythrocyte binding activity of PvTRAgs.** Histidine tagged PvTRAgs were incubated with (A) 1∶30 dilution of *P. vivax* infected patient sera alone (control), and with different dilutions (1∶10, 1∶100 and 1∶1000) of sera raised in rabbits or with (B) 1∶1000 dilution of rabbit antibodies alone (control), and with different dilutions (1∶10, 1∶50 and 1∶100) of *P. vivax* infected patients’ sera. Pre-incubated mixture was then allowed to react with erythrocytes and binding was detected by monoclonal anti-His_6_ antibody as described in text. Binding of PvTRAgs in the absence of patients’ sera or rabbit antibody (PBS) is considered as 100%. Error bar indicates the standard deviation of mean of percentage of binding from three experiments.
(DOCX)

Table S1 **Mean Fluorescence Intensity values showing binding of different PvTRAgs with reticulocytes and normocytes.** The MFI values have been normalized by nonspecific thioredoxin binding. Results are arithmetic means of three separate experiments. Values are mean ± standard deviation. The difference of MFI between binders and non-binders was statistically significant (P<0.05).
(DOCX)
