# Supplementary figures and images for: Erythrocyte Binding Activity Displayed by a Selective Group of Plasmodium vivax Tryptophan Rich Antigens Is Inhibited by Patients’ Antibodies
Source: PLoS One. 2012 Dec 6;7(12):e50754. doi: 10.1371/journal.pone.0050754 (PMC3516511; doi:10.1371/journal.pone.0050754)

Fig S1

Tyagi & Sharma


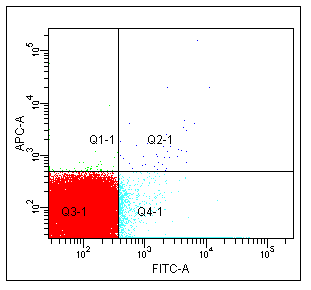

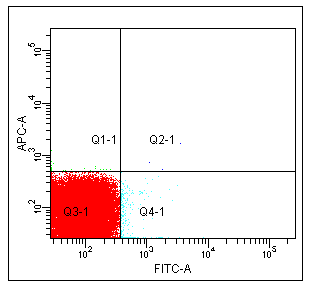

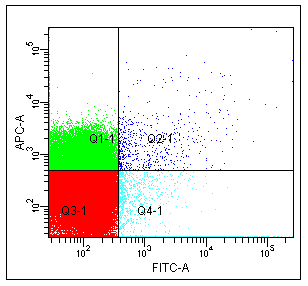

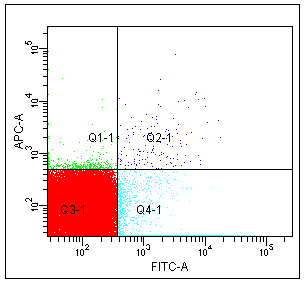

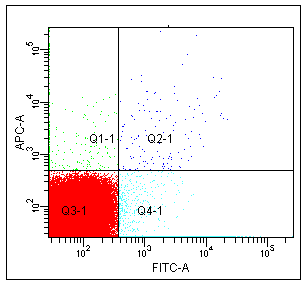

Supplement: Figure S1 — Dot plots for binding of PvTRAgs to erythrocytes. Erythrocytes were incubated with 1 µM of recombinant PvTRAgs and then labeled with an anti-penta-His mAb Alexa Fluor 647 conjugate and Thiazole Orange. A: Unstained, B: Background control without proteins, C: PvTRAg38, D: PvTRAg40 and E: Thioredoxin (DOCX) [file pone.0050754.s001.docx]
