# Supplementary material for: Erythrocyte Binding Activity Displayed by a Selective Group of Plasmodium vivax Tryptophan Rich Antigens Is Inhibited by Patients’ Antibodies
Source: PLoS One. 2012 Dec 6;7(12):e50754. doi: 10.1371/journal.pone.0050754 (PMC3516511; doi:10.1371/journal.pone.0050754)
Supplement: Figure S2 — Cell-ELISA based competition between rabbit antibodies and P.vivax patients’ sera for inhibition of erythrocyte binding activity of PvTRAgs. Histidine tagged PvTRAgs were incubated with (A) 1∶30 dilution of P. vivax infected patient sera alone (control), and with different dilutions (1∶10, 1∶100 and 1∶1000) of sera raised in rabbits or with (B) 1∶1000 dilution of rabbit antibodies alone (control), and with different dilutions (1∶10, 1∶50 and 1∶100) of P. vivax infected patients’ sera. Pre-incubated mixture was then allowed to react with erythrocytes and binding was detected by monoclonal anti-His6 antibody as described in text. Binding of PvTRAgs in the absence of patients’ sera or rabbit antibody (PBS) is considered as 100%. Error bar indicates the standard deviation of mean of percentage of binding from three experiments. (DOCX) [file pone.0050754.s002.docx]

Fig S2

Tyagi & Sharma
