# Supplementary material for: Erythrocyte Binding Activity Displayed by a Selective Group of Plasmodium vivax Tryptophan Rich Antigens Is Inhibited by Patients’ Antibodies
Source: PLoS One. 2012 Dec 6;7(12):e50754. doi: 10.1371/journal.pone.0050754 (PMC3516511; doi:10.1371/journal.pone.0050754)
Supplement: Table S1 — Mean Fluorescence Intensity values showing binding of different PvTRAgs with reticulocytes and normocytes. The MFI values have been normalized by nonspecific thioredoxin binding. Results are arithmetic means of three separate experiments. Values are mean ± standard deviation. The difference of MFI between binders and non-binders was statistically significant (P<0.05). (DOCX) [file pone.0050754.s003.docx]

**Supplementary Table1**: Mean Fluorescence Intensity values showing binding of different PvTRAgs with reticulocytes and normocytes.

| Antigens | Mean Fluorescence Intensity (MFI) | |
| --- | --- | --- |
|  | Reticulocytes | Normocytes |
| PvTRAg | 62±9 | 65±14 |
| \| PvTRAg80.6 \| \| --- \| | 9±2 | 8±3 |
| PvTRAg35.2 | 78±12 | 76±11 |
| \| PvTRAg40 \| \| --- \| | 11±3 | 7±3 |
| \| PvTRAg69.4 \| \| --- \| | 54±14 | 51±15 |
| \| PvTRAg38 \| \| --- \| | 85±12 | 81±18 |
| PvTRAg43.1 | 13±4 | 9±2 |
| \| PvTRAg53.7 \| \| --- \| | 11±3 | 7±2 |
| \| PvTRAg33.5 \| \| --- \| | 71±17 | 82±12 |
| \| PvTRAg39.9 \| \| --- \| | 8±2 | 12±3 |
| PvTRAg32.4 | 15±2 | 8±4 |
| PvATRAg74 | 59±17 | 55±16 |
| \| PvTARAg55 \| \| --- \| | 9±4 | 11±4 |
| \| PvTRAg42.9 \| \| --- \| | 10±3 | 12±3 |
| PvTRAg39.8 | 8±4 | 5±2 |

The MFI values have been normalised by nonspecific thioredoxin binding. Results are arithmetic means of three separate experiments. Values are mean ± standard deviation. The difference of MFI between binders and non-binders was statistically significant (P<0.05).
